# 960. Impact of Laser Scar Program at a Safety Net Hospital

**DOI:** 10.1093/jbcr/irag033.538

**Published:** 2026-04-06

**Authors:** Grace S Saglimbeni, Arpana Jain, Paul Kang, Erica Whetten, Philomene Spadafore, Suzanne C Osborn, Karen J Richey, Kevin N Foster

**Affiliations:** Creighton University School of Medicine, Phoenix, AZ; Diane & Bruce Halle Arizona Burn Center - Valleywise Health, Phoenix, AZ; Creighton University School of Medicine, Phoenix, AZ; Valleywise Health Medical Center, Phoenix, AZ; Diane & Bruce Halle Arizona Burn Center - Valleywise Health, Phoenix, AZ; Diane & Bruce Halle Arizona Burn Center - Valleywise Health, Phoenix, AZ; Diane & Bruce Halle Arizona Burn Center - Valleywise Health, Phoenix, AZ; Diane & Bruce Halle Arizona Burn Center - Valleywise Health, Phoenix, AZ

## Abstract

**Introduction:**

Burn scars cause significant physical, functional, and psychosocial impairment. Safety-net hospitals serve vulnerable populations but often face barriers to implementing advanced therapies. Fractional CO2 laser treatment has been shown to improve scar outcomes; however, its feasibility in resource-limited settings remains unclear.

**Methods:**

We performed a single-center, retrospective chart review at a publicly funded county hospital. It includes adults who underwent laser scar ablation from 2019–2024. Exclusions criteria are pediatric patients (age less than 18 years), incarcerated individuals, and psychiatric inpatients. Data collected includes demographics, hospital and provider charges, institutional costs, no-show rates, and adverse outcomes. This study was approved by the institutional IRB.

**Results:**

This study included 88 patients who underwent fractional CO₂ laser scar ablation at a safety-net hospital between 2019–2024. The cohort had a mean age of 30.5 years, 55% were male, and 31% identified as Hispanic/Latino.

Regression analysis revealed no significant associations between race or ethnicity and hospital charges, provider charges, or institutional costs. Significant associations were observed for age, with patients 18–29 and 30–44 years incurring higher provider charges compared to those <18 years.

Breakeven analysis at a 90% reimbursement rate demonstrated positive contribution margins for both models. When only hospital charges were considered, the contribution margin was $54720.62 per patient, while the combined hospital plus provider charge model yielded $55342.79 per patient. In both models, breakeven was reached after five patients, after which additional treatments generated net positive margins.

**Conclusions:**

Fractional CO₂ laser treatment for burn scars can be feasibly implemented in a safety-net hospital. Financial modeling suggests the program reaches breakeven after a small patient volume and maintains positive margins thereafter. In addition to financial sustainability, this intervention has the potential to improve mobility, function, and quality of life in underserved populations.

**Applicability of Research to Practice:**

Demonstrating feasibility of a laser scar program in a safety-net hospital could guide broader adoption of advanced burn scar care in resource-limited settings and enhancing long-term outcomes for all patient populations.

**Funding for the study:**

College of Medicine Student Research award.

